# Chronodisruption: A Poorly Recognized Feature of CKD

**DOI:** 10.3390/toxins12030151

**Published:** 2020-02-28

**Authors:** Sol Carriazo, Adrián M Ramos, Ana B Sanz, Maria Dolores Sanchez-Niño, Mehmet Kanbay, Alberto Ortiz

**Affiliations:** 1 IIS-Fundacion Jimenez Diaz, Department of Medicine, Universidad Autonoma de Madrid, Fundacion Renal Iñigo Alvarez de Toledo-IRSIN, 28040 Madrid, Spain; sol.carriazo@quironsalud.es (S.C.); amramos@fjd.es (A.MR.); asanz@fjd.es (A.BS.); mdsanchez@fjd.es (M.D.S.-N.); 2 Red de Investigación Renal (REDINREN), 28040 Madrid, Spain; 3 Division of Nephrology, Department of Medicine, Koc University School of Medicine, 34010 Istanbul, Turkey; drkanbay@yahoo.com

**Keywords:** chronodisruption, chronodisruptor, circadian rhythm, internal clock, chronic kidney disease

## Abstract

Multiple physiological variables change over time in a predictable and repetitive manner, guided by molecular clocks that respond to external and internal clues and are coordinated by a central clock. The kidney is the site of one of the most active peripheral clocks. Biological rhythms, of which the best known are circadian rhythms, are required for normal physiology of the kidneys and other organs. Chronodisruption refers to the chronic disruption of circadian rhythms leading to disease. While there is evidence that circadian rhythms may be altered in kidney disease and that altered circadian rhythms may accelerate chronic kidney disease (CKD) progression, there is no comprehensive review on chronodisruption and chronodisruptors in CKD and its manifestations. Indeed, the term chronodisruption has been rarely applied to CKD despite chronodisruptors being potential therapeutic targets in CKD patients. We now discuss evidence for chronodisruption in CKD and the impact of chronodisruption on CKD manifestations, identify potential chronodisruptors, some of them uremic toxins, and their therapeutic implications, and discuss current unanswered questions on this topic.

## 1. Introduction: The Growing Global Health Burden of Chronic Kidney Disease

Chronic kidney disease (CKD) is currently defined as abnormalities of kidney structure or function, present for longer than 3 months, with implications for health [1]. The abnormalities of kidney structure or function may be recognized by several criteria. Just one of these criteria is enough to diagnose CKD. The most commonly used criteria are the ones that characterize CKD categories: An abnormal function defined by a decreased glomerular filtration rate (GFR, <60 mL/min/1.73 m^2^, that is, G categories G3–G5) or evidence of kidney damage such as albuminuria (albumin excretion rate ≥ 30 mg/24 h; urinary albumin creatinine ratio ≥ 30 mg/g, that is, A categories A2 or A3). As for the concept of “implications for health”, it reflects the fact that CKD is associated with an increased risk of all-cause or cardiovascular death, of CKD progression and of development of acute kidney injury (AKI). In this regard, the contribution of CKD to the global disease burden has increased sharply in recent decades. CKD is estimated to become the fifth global cause of death by 2040 and in countries with long life expectancies, it has been projected to become one of the two top causes of death before the end of the century [2,3]. The increasing contribution of CKD to the global burden of disease can be traced to several causes. On one hand, age-adjusted mortality for some key causes of death is actually decreasing. On the other, the longer life expectancy of the population and the increasing prevalence of risk factors for CKD such as obesity, diabetes and hypertension, together with the underdeveloped therapeutic armamentarium, are driving up the prevalence and impact of CKD. There is hope in the recent characterization of a dramatic nephroprotective impact of sodium-glucose transport protein 2 (SGLT2) inhibitors when added on top of renin angiotensin system (RAS) blockade for diabetic kidney disease and potentially other kidney diseases [4,5,6]. However, data from the hypertension field have clearly demonstrated that the availability of effective drugs is not enough, especially in polymedicated populations, where guidelines now emphasize measures to facilitate compliance [7]. In any case, the increasing burden of CKD at a time when other major causes of death are decreasing should be viewed in the context of the paucity of new therapeutic options that have become available in recent years, when compared, for example, with the cancer field [8]. This points towards major deficiencies in our understanding of the pathogenesis of CKD and of the pathophysiology of the CKD-associated increase in cardiovascular risk and premature aging. A key feature of advanced CKD is accumulation of uremic toxins that are no longer excreted by damaged kidneys, although in some instances increased toxin production also contributes to CKD manifestations [9,10,11]. However, this would not explain why there is already an increased risk of death when GFR is preserved, i.e., in patients in whom CKD is diagnosed because of abnormally high albuminuria yet GFR is still above 60 mL/min/1.73 m^2^. Additional pathogenic events have been recently identified in these patients, such as loss of the kidney production of the anti-aging factor Klotho [12]. A long-recognized feature of CKD is an alteration of well characterized circadian rhythms, including circadian changes in blood pressure and urine concentrating ability. The widespread use of 24 h ambulatory blood pressure monitoring has familiarized physicians with the concept of the sleep time dip of blood pressure and the lack of such dip in CKD patients: CKD patients are characteristically non-dippers [13]. However, the molecular basis of this altered blood pressure circadian rhythm and the existence of other altered rhythms as well as the consequences of these altered rhythms for CKD progression and CKD-associated morbidity and mortality are less well known. We now review the basics of internal clocks and circadian rhythms, the concept of chronodisruption and how this concept applies to CKD leading to the identification of kidney and central chronodisruptors characteristic of the CKD situation and how this may change our approach to CKD management.

## 2. Biological Rhythms

Exposure to periodic environmental changes during evolution is thought to have driven the development of adaptive biological rhythms of which the best known are the circadian rhythms, which have a period length of around 24 h. However, there are also ultradian rhythms (>24 h) and infradian rhythms (<24 h) [14,15]. Biological rhythms allow the adaptation to changing environments, from the light-night cycle, to the seasons or feed-fast cycles. However, current 24/7 lifestyles dim the environmental differences between day and night, resulting in weak zeitgebers (weak day light, absence of darkness during night, constant environmental temperature, sedentarism and frequent snacking), which may impair the circadian system [16].

**Figure 1:**
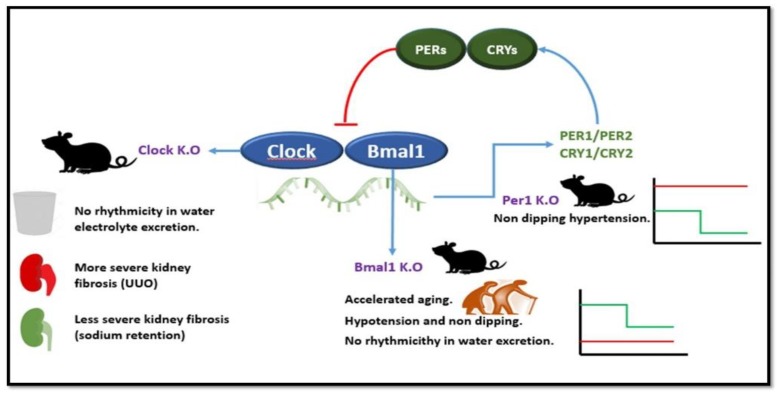
Canonical clock genes and the basic regulatory loop: impact on the kidney of genetic defects. In the most basic regulatory loop, *Clock* and *Bmal1* are transcription factors that promote *Cry* and *Per* gene expression, and Cry and Per proteins, in turn, suppress *Clock*/*Bmal1* induction of their Cry and Per transcription. Genetic disruption of some canonical clock genes has yielded renal-hypertension phenotypes as illustrated above for *Clock*, *Bmal1*, and *Per1* in mice. *Clock* KO mice display loss of water and electrolyte excretion rhythmicity as well as differential responses to induction of kidney fibrosis, which appears specific of the driver of fibrosis (worse unilateral ureteral obstruction (UUO)-induced fibrosis but milder sodium overload-induced fibrosis). *Bmal1* KO mice display accelerated aging, loss of rhythmicity of water excretion as well as non-dipping hypotension (red line) as compared to the normal blood pressure circadian rhythm (green line). *Per1* KO mice display non-dipping hypertension (red line) as compared to the normal blood pressure circadian rhythm (green line).

The central circadian clock lies in the suprachiasmatic nucleus in the anterior hypothalamus and coordinates peripheral clocks, including the kidney circadian clock which, in turn, coordinate local physiologic functions with patterns of activity and/or feeding [17]. Several signals contribute to coordinate peripheral circadian rhythms, including hormone secretion (e.g., production of the melatonin hormone by the pineal gland during nighttime, circadian production of aldosterone), neuronal activity (including physical activity and feeding) and body temperature. In addition, canonical clock genes (e.g., *Clock*, *Bmal1*, *Rev-erbα*, *Cry1*, *Cry2*, *Per1*, *Per2*) are expressed and/or active in a cyclical manner within cells, driving cell autonomous circadian rhythms [14,15]. In the most basic regulatory loop, *Clock* and *Bmal1* are transcription factors that promote *Cry* and *Per* gene expression, and *Cry* and *Per* in turn suppress *Clock/Bmal1* induction of their own transcription [18] (Figure 1). On top of this basic regulatory loop, associated elements account for the circadian regulation of 13% of kidney expressed genes. Furthermore, posttranslational modifications (e.g., phosphorylation, acetylation) are also responsible for circadian changes in protein activity. Functional circadian molecular clockwork evolves in the late fetal and early postnatal kidney. During the nursing period, oscillations are entrained by nutritional cues [19].

Kidney function has circadian rhythms (Table 1). The amplitude of circadian oscillations in GFR and renal plasma flow are around 50%, while water and electrolyte (sodium, potassium, calcium, magnesium, and phosphate) excretion may be several fold higher during the active phase and this is paralleled by circadian changes in kidney oxygenation and the corticomedullary interstitial osmolarity gradient and in the expression of genes involved in its regulation (e.g., vasopressin receptors V1aR, V2R, urea transporter UT-A2 and water channel Aqp2) [14]. Changes in kidney oxygenation modulate HIF-1α activation and erythropoietin levels, which display an amplitude of more than 10-fold under constant darkness and normoxia in mice [15]. Blood pressure peaks early in the beginning of the active period of both diurnal and nocturnal animals [20]. Molecular clocks regulate sodium balance, sympathetic function and vascular tone, all contributing to blood pressure regulation. Altered kidney circadian rhythms have been associated with the development of hypertension, chronic kidney disease, and kidney stones (reviewed in [14]).

Insights into the circadian regulation of kidney functions is derived from genetic defects in clock genes [14] (Figure 1). Thus, *Per1* KO mice develop non-dipping hypertension under conditions of sodium retention while *Clock* KO mice lose the circadian rhythmicity in urinary water and electrolyte excretion and develop more severe kidney fibrosis upon ureteral obstruction but were protected from kidney fibrosis driven by sodium retention conditions [14]. Additionally, *Clock* mutants had some features suggesting increased severity of adenine-induced CKD, such as higher blood pressure and expression as some gelatinase genes, but there were no differences in kidney fibrosis or serum creatinine [21]. *Bmal1* KO mice develop accelerated aging, hypotension and a non-dipping blood pressure pattern and lose the circadian variations in interstitial medullary osmolarity suggesting a role of circadian clocks in the control of urine volume beyond dietary clues [14,22]. Kidneys from conditional nephron-specific *Bmal1* deletion mice exhibited a decrease in NAD+-to-NADH ratio, increase in plasma urea and creatinine and a reduced capacity of the kidney to secrete anionic drugs (furosemide) paralleled by changes in the expression of tubule transporters such as organic anion transporter 3 (SLC22a8) [23]. Na+-H+ exchanger 3 (NHE3) activity also has rhythmic oscillations causing daily fluctuations in Na+ and water transport of the proximal tubule cell.

## 3. Concept of Chronodisruption

**Figure 2:**
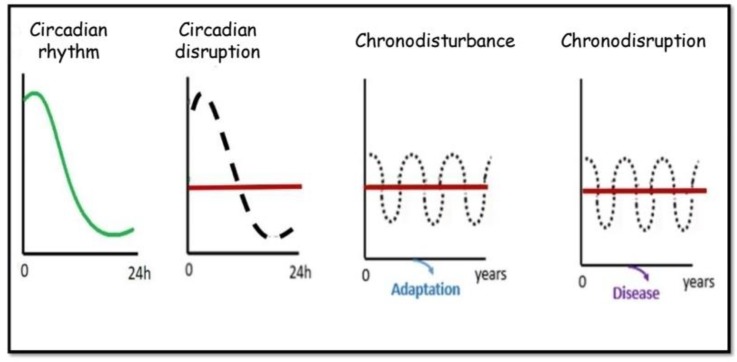
Concepts of circadian disruption, chronodisturbance and chronodisruption. As compared to a normal circadian rhythm, circadian disruptions are characterized by altered circadian rhythm that may be short or long lived. Chronodisturbance is a chronic disruption of circadian rhythms that somehow leads to adaptive phenomena that limit its negative impact. Chronodisruption is a chronic disruption of circadian rhythms that results in disease. Chronodisruptors (not shown) are the factors driving chronodisruption. The normal circadian rhythm is shown as a green line in the left panel and as a discontinuous line in the other panels. A red line represents the altered circadian rhythm in the three left panels. Please note the different timelines shown in the horizontal axis, with chronodisturbance and chronodisruption implying chronicity.

The concept of chronodisruption was coined in 2003 by Thomas C. Erren, Russel J. Reiter and Claus Piekarski from the University of Cologne [24] (Figure 2). The term was meant to go beyond the concept of chronodisturbance, a general term they proposed to refer to modulations of rhythms over time that are not necessarily deleterious since physiological compensations may prevent the development of chronic disease resulting from altered rhythms. Chronodisturbance itself was a conceptual leap from more common concepts such as “circadian disruption” or “disruption of circadian rhythms” that suggest that rhythms over 24 h can become desynchronized and that this may have adverse health effects, since these common terms may be more limited in time scope than chronodisturbance which may have a decade scope. Thus, circadian disruption may be caused by travel across several time zones, however, within a limited period of time within this new time zone, adaptation of the circadian rhythms to the new time zone occurs and there are no long-term consequences. By contrast, chronic work in night shifts will lead to chronodisturbance, that is to persistent desynchronization between time and activity. In 2009, they further elaborated on the chronodisruption concept, stating that “chronodisruption can be understood as a critical loss of time order, i.e., a disorder or chaos of an otherwise physiological timing at different organizational levels, including the gene expression levels in individual cells” and thus, it is “a breakdown of phasing internal biological systems appropriately relative to the external, i.e., environmental changes, which leads to chronobiological disorders” [25]. Following with the chronic night shift example, this would be considered chronodisturbance as long as there are no adverse consequences for health, and chronodisruption if this leads to adverse consequences for health. Furthermore, they characterized chronodisruptors as “exogenous and endogenous exposures or effectors which are chronobiologically active and can thus disrupt the timing and order, i.e., the temporal organization of physiologic functions and hierarchies” [25]. A clear example of a chronodisruptor is the use of artificial light or backlit screens during the night. They additionally proposed that assessment of melatonin levels in saliva, urine and blood may be a robust biomarker of chronodisruption. While in some fields the concept was immediately grasped (In 2007, the International Agency for Research on Cancer classified shift-work that involves circadian disruption as probably carcinogenic to humans), it was not until 2013 that the term chronodisruption was used in the context of CKD [26] and only in 2019 was a second manuscript published on the topic [27].

While this is surprising given the chronic nature of CKD, its similarities with aging and the widely known fact that circadian rhythms may be disturbed in CKD, it does not mean that the nephrological community is not aware of disruption of circadian rhythms in CKD. Indeed, very active research is going on as attested by recent reviews [14,15,26,28,29]. However, CKD researchers may benefit from a wider use of the terms and concepts of chronodisruption and chronodisruptor. Thus, the mere concept of chronodisruptor may facilitate the search of chronodisruptors involved in CKD manifestations. These may potentially be abnormal levels of uremic toxins or abnormally low levels of uremia-related factors, among others.

## 4. Chronodisruption in CKD

Several alterations of circadian rhythms are well characterized in CKD patients and there is accumulating evidence that at least some of them may adversely affect health, thus fulfilling criteria to be considered chronodisruption. These include disordered sleep, non-dipping hypertension, failure to properly concentrate urine at night and the circadian pattern of proteinuria in patients with nephrotic syndrome: Peak protein excretion occurs at around 16.00 h and the nadir at 03.00 h and is independent of GFR [14]. Ultradian rhythms have also been described in CKD. For example, in patients with end-stage renal disease treated with hemodialysis, blood pressure varies seasonally, with higher values in the winter and lower values in the summer [30]. However, this pattern has not been directly compared to that of non-CKD individuals. Of the diverse altered circadian rhythms in CKD, the ones best characterized with adverse consequences for health, meeting the criterion to define chronodisruption are disordered sleep and non-dipping hypertension and will be discussed more extensively.

Sleep timing, quality and/or duration are frequently disturbed in CKD patients and this can be reproduced by subtotal nephrectomy in rats [26] or in mice with adenine-induced CKD [21]. In patients with mild to moderate CKD, lower eGFR was associated with shorter sleep duration (−1.1 mL/min/1.73 m^2^ per hour less sleep), greater sleep fragmentation (−2.6 mL/min/1.73 m^2^ per 10% higher fragmentation) and later timing of sleep (−0.9 mL min/1.73 m^2^ per hour later). Higher proteinuria was also associated with greater sleep fragmentation (approximately 28% higher per 10% higher fragmentation) [31]. However, from these studies, potential causality and direction of the association is unclear, since CKD may cause chronodisruption but chronodisruption may theoretically lead to CKD progression. The nocturnal melatonin peak appears to be preserved just in nocturnal hemodialysis patients but not in patients on other dialysis modalities. In this regard, exogenous melatonin may improve intrarenal renin angiotensin system activation and renal injury in experimental CKD [32]. Specific conditions associated to CKD may contribute to disrupted sleep patterns. These include nocturia elated to decreased urine concentration capacity and obstructive sleep apnea. The prevalence of obstructive sleep apnea increases as kidney function declines and is higher among patients with ESRD. obstructive sleep apnea may contribute to higher nocturnal blood pressure and to pulmonary hypertension and these may improve on continuous positive airway pressure (CPAP) [33,34,35].

In CKD patients, the prevalence of reverse dipping (night-time blood pressure peak) for systolic blood pressure and episodes of hypotension during daytime is doubled, independently of blood pressure control [36]. Uninephrectomy by itself interfered with blood pressure rhythms. Albuminuria in hypertensive patients is also accompanied by quantitatively striking higher nighttime systolic blood pressure, particularly in patients with diabetes with very high albuminuria and low eGFR [37]. Although studies regarding causality are needed, this observation may point out to a CKD A2/A3-dependent altered clock: That is, albuminuria itself may potentially be a chronodisruptor, even when global kidney function (GFR) is preserved, on top of any potential chronodisruptor activity of uremic toxins that accumulate when GFR falls. Further supporting a potential role of albuminuria itself, in minimal change nephrotic syndrome patients with overall preserved GFR (around 75 mL/min/1.73 m^2^), sleeping/waking systolic and diastolic blood pressure ratios were higher than in healthy controls and this was reversed by remission of proteinuria [38].

Non-dipping is a recognized cardiovascular risk factor. In the general population, there is a linear relationship between the nocturnal decline in blood pressure and cardiovascular mortality. On average, each 5% decrease in the decline in nocturnal systolic/diastolic blood pressure was associated with an approximately 20% greater risk of cardiovascular mortality and this was observed even when 24-h blood pressure values were within the normal range (average 118/69 mmHg), diminished nocturnal decreases [39]. In CKD patients this may be magnified, as they have higher systolic blood pressure during the night-time and greater prevalence of non-dipping. Indeed, nocturnal systolic blood pressure correlated more strongly with cardiac organ damage [40]. In hemodialysis patients, increased short-term nighttime pulse pressure variability but not ambulatory blood pressure levels were significantly predictive of long-term all-cause mortality [41].

Several individual contributors to the circadian regulation of blood pressure have been identified and these include local kidney molecular clocks, whose local expression may be potentially altered by kidney disease mediators. Thus, *Bmal1* deficiency in juxtaglomerular renin-secreting granular cells resulted in polyuria, changes in the circadian rhythm of urinary sodium excretion, increased GFR, and lower plasma aldosterone levels and lower blood pressure [42]. The sodium-chloride cotransporter (NCC, SLC12A3) in distal convoluted tubules contributes to sodium balance and blood pressure regulation. Disturbing this rhythm induces “nondipping” blood pressure. Both mineralocorticoids and glucocorticoids regulate NCC activity. Mineralocorticoid receptor activation maintains the NCC protein pool while glucocorticoid receptor activation regulates NCC phosphorylation and the diurnal rhythm of NCC activity [43]. ATP1B1 encodes the β1 subunit of the Na^+^/K^+^-ATPase. Atp1b1 mRNA and protein levels in mouse kidney have a circadian rhythm that was antiphasic to the blood pressure rhythm. In *Dec1*-deficient mice, kidney Atp1b1 expression was increased and blood pressure was lower. In contrast, in *Clock*-mutant mice, Atp1b1 expression was low and blood pressure high [44]. The expression of both NCC and ATP1B1 is altered in kidney injury, potentially linking kidney injury to an altered expression of kidney circadian genes regulating blood pressure [45,46].

The location of disrupted timekeeping in CKD merits further study. In murine adenine-induced CKD, in vivo disrupted timekeeping could be dissociated in vitro into a suprachiasmatic nucleus pacing, which remained uncompromised, and a kidney clock that became a less robust circadian oscillator with a longer period, suggesting that the kidney contributes to overall circadian timekeeping and that there is local kidney disruption of circadian rhythms during CKD [47]. By contrast, in vivo exploration of mice with adenine-induced CKD disclosed low amplitude PER2:luciferase rhythms in their central suprachiasmatic nucleus circadian clock and in intact kidney, liver, and submandibular gland, as well as altered expression patterns of circadian genes including canonical clock genes and kidney genes such as Hif, Aqp2, and V2r [21]. Overall, these results point to interference of peripheral clocks with the central clock in CKD.

Failure to properly concentrate urine at night may further aggravate CKD-associated sleep disruption through nocturia. However, there are potentially more severe consequences. Thus, improper water excretion will promote the secretion of antidiuretic hormone (vasopressin, ADH). There is increasing evidence that overactivation of ADH may be detrimental. Specifically, the vasopressin 2 receptor (V2R) blocker tolvaptan slows the progression of autosomal dominant polycystic kidney disease (ADPKD) [48]. While this was initially thought to be related to kidney cyst specific intracellular signaling events, an adverse impact of ADH on glomerular hyperfiltration was later identified that may be a universal driver of CKD progression, not limited to ADPKD [49,50]. In this regard, circulating copeptin levels provide a better understanding of ADH activation that measuring ADH itself, which is short lived. Serum copeptin is increased in hypertension, CKD and cardiovascular disease, and ADH activation of V1R and/or V2R may be detrimental to the kidney and the cardiovascular system [51].

The altered circadian pattern of proteinuria may impact the assessment of the severity or proteinuria when different timed urine samples are assessed (12 h vs. 24 h vs. point collections), but whether this leads to any health consequence is currently unclear.

CKD has a bidirectional relationship with aging. On one hand, aging is associated with a progressive decrease in GFR. On the other, CKD causes accelerated aging and some of the factors responsible for this phenotype, such as decreased production of the anti-aging factor Klotho have been identified, as discussed below. Interestingly, aging is associated with altered central and peripheral circadian rhythms, and the sleep–wake cycle [52], leading to a phase advance, rhythm fragmentation and flattening [53]. This may in part be offset by regular physical activity [52]. Given the close association of CKD with aging, further studies are required that explore to what extent the age-associated loss of renal function contributes to age-associated circadian rhythm abnormalities and age-associated organ dysfunction and disease.

## 5. Chronodisruptors as Therapeutic Targets in CKD

A PubMed search for “chronodisruptors” in January 2020 resulted in only 5 hits, none of them related to CKD. This may relate to both limited understanding of chronodisruptors as with limited use of the term.

Identifying and targeting chronodisruptors may identify novel approaches to the prevention and therapy of CKD. Potential chronodisruptors include diet, the light–dark cycle, inflammatory mediators, uremic toxins, HIF abnormalities, and physical inactivity. We will briefly discuss examples of all of these (summarized in Table 2). While diet, light clues and inflammation may be active at all stages of CKD, even before GFR decreases, accumulation or uremic toxins would be expected to be active only after significant decrease of GFR has taken place, i.e., after significant loss of kidney mass.

### 5.1. Dietary Clues

There is some evidence that dietary lipids and sodium may behave as chronodisruptors and, more specifically, that salt may be a chronodisruptor in CKD. Indeed, salt loading aggravates the inverse relationship between melatonin secretion, assessed as urinary levels of its metabolite 6-sulfatoxymelatonin (aMT6s) and albuminuria in CKD patients [54]. High salt feeding led to region-specific alterations in circadian clock components within the kidney and caused a 5.5-h phase delay in the peak expression of *Bmal1* and suppressed *Cry1* and *Per2* expression in the renal inner medulla, but not the renal cortex, of control rats. The phase delay in *Bmal1* expression appears to be mediated by endothelin-1 because this phenomenon was not observed in endothelin receptor B (ETB)-deficient rats. Thus, high salt feeding leads to intrarenal circadian dyssynchrony in part through activation of ETB receptors within the renal inner medulla [55]. There is less information on the molecular mechanisms engaged by dietary lipids to influence circadian kidney rhythms. One possibility is through epigenetic regulation of gene expression. Thus, dietary lipids modulate the expression of miR-107, a miRNA that regulates the circadian system [56].

An area of research is focused on altering circadian rhythms by time-related dietary approaches (chrononutrition) or pharmacological substances (chronobiotics) [57]. In a randomized clinical trial, short chronotype-adjusted diet was more effective than the traditional hypocaloric diet in decreasing BMI, and waist circumference [58]. In a further trial, eating late was associated with decreased resting-energy expenditure, decreased fasting carbohydrate oxidation, decreased glucose tolerance and blunted daily profile in free cortisol concentrations [59]. In this regard, it is widely recognized that chronodisruption and mistimed eating have deleterious effects on metabolic health that may exceed those of eating an unbalanced diet, during the normal active phase [60]. How CKD may affect these relationships and to what extent chronotype-adjusted diets may provide any advantages to CKD patients is, at this point, unclear.

Diet may also influence the gut microbiota. Gut bacteria modulate host rhythms via microbial metabolites such as butyrate and others, and amines and disturbed microbiome rhythms have been proposed to at least partially contribute to an increased risk of obesity and metabolic syndrome associated with chronodisruption [61]. Although there is little information on microbiota and chronodisruption in CKD, both obesity and metabolic syndrome increase the risk of CKD. Conversely, CKD has been associated with altered microbiota patterns and metabolites accumulated in CKD may modulate the gut microbiota and butyrate production [62,63,64].

### 5.2. Light Clues

In June 2019, a working group convened by the International Agency for Research on Cancer (IARC) concluded that “night shift work” is probably carcinogenic to humans and considered a Group 2A carcinogen [65]. There is very little information on night shift work and CKD. However, in a Korean study, the risk of CKD was two-fold higher in female shift workers than in female non-shift workers, although there were no differences in males [66]. In experimental animals, maternal chronic photoperiod shifting during gestation led to kidney gene expression changes in the offsprings, including the expression of sodium handling genes subject to circadian rhythms, and higher blood pressure values [27].

### 5.3. Kidney Inflammation

Kidney inflammation is a feature of both AKI and CKD. TWEAK is a proinflammatory cytokine of the TNF superfamily that promotes AKI and CKD [67,68]. A key feature of the TWEAK cytokine is that, contrary to TNF, it recruits the NIK-mediated, non-canonical pathway for activation of the NFκB transcription factor in kidney cells on top of the canonical pathway for NFκB activation [69,70,71,72,73]. NFκB is a key proinflammatory transcription factor that also downregulates kidney protective molecules [74]. Non-canonical NFκB is characterized by the nuclear translocation of RelB/NFκB2 p52 heterodimers [75]. Interestingly, the RelB subunit of NFκB directly binds *BMAL1* and acts as a negative regulator of circadian gene expression [76]. TWEAK also downregulates the kidney production of Klotho, an antiaging factor that is mainly expressed in the kidney, thus, potentially contributing to the accelerated aging of CKD [77,78]. Although the decrease in Klotho is mediated by the canonical NFκB pathway, it is nonetheless integrated within the cell response to TWEAK characterized by downregulation of tissue protective factors, as is a decrease in the mitochondrial biogenesis master regulator PGC1α [79,80]. In his regard, RelB also couples with the bioenergy NAD (+) sensor sirtuin 1 (SIRT1) to modulate cell metabolism and mitochondrial bioenergetics [81].

Kidney fibrosis sis very tightly linked to inflammation. In this regard, Smad3, a key signaling effector for the profibrotic cytokine TGFβ1, has circadian expression and modulates the expression of circadian rhythm genes such as *Dec1*, *Dec2*, and *Per1* [82].

### 5.4. Uremic Toxins

A key feature of advanced CKD is the accumulation of uremic retention solutes, molecules usually excreted by the kidneys that accumulate in the circulation when GFR decreases [11]. Some of these uremic retention solutes have a clear adverse impact on pathophysiological processes, promoting CKD progression and manifestations, they are the so-called uremic toxins. When kidneys fail, renal function is replaced by dialysis or eventually by a kidney graft. Unfortunately, while dialysis prevents acute uremic death, it provides only a very limited capacity to clear uremic toxins, especially those of gut origin that circulate bound to serum proteins, which may be of special interest from the point of view of chronodisruption. Thus, several gut-derived uremic toxins bind and activate the Aryl Hydrocarbon Receptor (AhR). These include uremic toxins derived from tryptophan, some of gut microbiota origin, such as indolic uremic toxins (indoxyl sulfate, indole-3 acetic acid, and indoxyl-β-d-glucuronide) and uremic toxins from the kynurenine pathway (kynurenine, kynurenic acid, anthranilic acid, 3-hydroxykynurenine, 3-hydroxyanthranilic acid, and quinolinic acid) [83,84]. Interestingly, AhR exhibits a rhythmic expression and time-dependent sensitivity to activation by AhR agonists and in response to at least some ligands, AhR forms a heterodimer with *Bmal1* and inhibits *Clock*/*Bmal1* activity, modulating amplitude and phase of rhythms in circadian clock genes [85,86]. In this regard, AhR deficiency enhanced behavioral responses to changes in the light–dark cycle, increased rhythmic amplitude of circadian clock genes in the liver, and altered glucose and insulin rhythms [86].

Kidney proximal tubule cells sense elevated endogenous, gut microbiome-derived, uremic retention solutes which elicit a compensatory response consisting of up-regulating the organic anion transporter-1 (OAT1), thus increasing metabolite secretion in urine [87]. This was clearly illustrated for indoxyl sulfate which induced OAT1 expression via AhR and EGFR signaling, controlled by miR-223 [87]. AhR protein expression was additionally positively associated with plasma levels of another indolic uremic toxin, indole-3 acetic acid (IAA) [88]. IAA is responsible for some adverse effects potentially related to the increased cardiovascular risk of CKD patients, such as increasing the expression of tissue factor in human vascular cells via the AhR [89]. However, up to now it is unknown to what extent the circadian expression of AhR is disrupted in CKD, what role might uremic toxins and the microbiota have in this phenomenon and what the consequences in any alterations in this system circadian regulation might be for CKD patients.

### 5.5. Disrupted HIF Activation and EPO Production

Hypoxia-inducible factor (HIF) are a family of transcription factors that protect from hypoxia both at the local, autocrine/paracrine level and by driving erythropoietin production, also through an endocrine mediator of kidney origin. Thus, the kidney has the lowest pO_2_ in the body, a consequence of the existence of two consecutive capillary networks (glomerular and peritubular) and of the high metabolic rate of tubular cells which spend huge amounts of energy in recovering filtered molecules. This is the likely reason for the kidney location of erythropoietin-producing cells, a key defense mechanism against hypoxia that modulates hemoglobin availability and, thus, oxygen transport capacity by red blood cells.

The expression of a key HIF protein, HIF1α, is under circadian rhythm control. CRY1 reduces HIF-1α half-life and HIF binding to target gene promoters and abrogation of CRY1/2 stabilized HIF1α in response to hypoxia [90] while PER2 activates HIF-1α and facilitates its recruitment to promoter regions of its downstream genes. HIF-1α activation by PER2 was related to keeping the asparagine residue at position 803 of HIF-1α (HIF-1α N803) unhydroxylated by hypoxic stimulation in the absence of changes in HIF-1α protein levels [91]. In murine heart ischemia, *Per2* was required for Hif-1α stabilization [92]. This may be exploited therapeutically. Thus, *Per2* stabilization through adenosine activation of Adora2b or by exposure to intense light modified HIF-dependent cardiac metabolism, resulting in the transcriptional induction of glycolytic enzymes and *Per2*-dependent protection from ischemia [92]. So far, no such experiments have been reported for kidney disease. By contrast, BMAL1 deficiency increased HIF1α protein levels under hypoxic conditions. Induction of clock and HIF1α target genes in response to strenuous exercise varied according to the time of day in wild-type mice. Thus, interactions between circadian and HIF pathways influence metabolic adaptation to hypoxia [93].

Circadian transgenic zebrafish cells simulating a repressed or an overstimulated circadian clock, resulted in altered gene transcription levels of oxygen-regulated genes such as *EPO* and altered the hypoxia-induced increase in Hif-1α protein concentration. The amount of Hif-1α protein accumulated during the hypoxic response depended on the time of the day, with one maximum during the light phase and a second one during the dark phase [94].

The positive effects of HIF prolyl hydroxylase inhibitors (that is, HIF activators) over anemia and other cardiovascular risk parameters in CKD patients [95] raises the possibility that downregulation of HIF activation righter than loss of renal mass is a key driver of uremic anemia and may allow the exploration of the chronodisruption impact of uremic anemia itself.

### 5.6. Physical Inactivity

Both the drivers (e.g., obesity) and consequences (e.g., anemia, cardiovascular disease, malnutrition) of CKD may be associated to physical inactivity and this may act as a chronodisruptor. The impact of regular physical activity on kidney functions circadian misalignment should be studied, since regular endurance exercise appears to entrain peripheral clocks in muscle and heart [52].

### 5.7. Integration of Several Chronodisruptors

It is likely that the end result of the impact of several chronodisruptors relates to the integration of the different signaling events. In this regard, there is evidence that chronodisruptors potentially associated with CKD interact between them. Thus, RelB directly binds to the AhR and AhR interacts with dietary clues [81,96]. AhR-deficient mice are protected from high fat diet-induced disruption in metabolic rhythms, exhibiting enhanced insulin sensitivity and glucose tolerance [96].

## 6. The Way Forward

Table 3 summarizes some key answered questions regarding chronodisruption, chronodisruptors and CKD. A key to the clinical translation of the current state of knowledge regarding chronodisruption in CKD, beyond preventing and treating CKD itself, is to identify targetable chronodisruptors.

An issue frequently overlooked by researchers is that the most common laboratory animals used to study kidney disease are rats and mice, which are nocturnal animals. Thus, essentially all experiments are performed during their inactive period and manipulation during this period risks creating chronodisruption which may have an unknown impact on experimental results [20]. This emphasizes the need for human studies. However, clinical research into CKD-related chronodisruption would require easy access to non-invasive techniques that allow monitoring of biological rhythms beyond blood pressure. Wrist skin temperature has been proposed as a new index for evaluating circadian system status [97]. Development of chronodisruption scores [98] and computational model of the renal circadian clock [99] would also facilitate clinical research. Longitudinal studies and ideally, interventional trials, would provide information on the causality and direction in the clinical association of disturbed sleep (a likely manifestation of chronodisruption) and CKD. In this regard, in a prospective cohort study of over 4000 participants from the Nurses’ Health Study, shorter sleep duration was prospectively and independently associated with faster decline in renal function [100].

Chronopharmacology studies how biological rhythms influence pharmacokinetics, pharmacodynamics, and toxicity, and determines whether time-of-day administration modifies the pharmacological characteristics of the drug. Chronotherapy applies chronopharmacological studies to clinical treatments, determining the best biological time for dosing [101]. Well known examples in CKD patients include phosphate binders. In addition, there is a school of thought supported by meta-analyses results and clinical trials emphasizing the benefits of nighttime administration of anti-hypertensive medication [14].

In a recent clinical trial in hypertensive patients without CKD, ingestion of at least one blood pressure-lowering medication at bedtime resulted in improved ambulatory blood pressure control with a significant further decrease of asleep blood pressure and reduced risk of incident CKD than early morning administration [102].

While this may be initially viewed as CKD prevention, it is likely that it may additionally represent slowing of CKD progression, Thus, current diagnostic criteria for CKD are late events and patients who progressed to meet the diagnostic criteria for CKD during the trial likely had baseline subclinical CKD, maybe as cause of hypertension [103]. New upcoming drugs may also benefit from chronopharmacology studies. Thus, HIF activators were recently approved for clinical use in China and are expected to be soon available worldwide to treat uremic anemia [104]. Whether chronopharmacology may optimize timing of administration is currently unknown. Finally, cardiovascular and nephroprotective effects have been described for melatonin [105].

## Figures and Tables

**Table 1:** Some examples of kidney functions which have circadian rhythms.

Glomeruli	Circulation and Interstitial	Tubular
Glomerular filtration rate	Renal plasma flow	Water and electrolyte (sodium, potassium, calcium, magnesium, phosphate) excretion and corticomedullary interstitial osmolarity gradient
	Kidney oxygenation and erythropoietin production	H^+^ excretion

**Table 2:** Examples of potential chronodisruptors in chronic kidney disease (CKD) patients.

Diet	Other Lifestyle Factors	Endogenous Factors
Dietary components, e.g., sodium	Night shift work	Gut microbiota and microbiota-associated uremic toxins
Mistimed eating		Kidney inflammation, non-canonical NFκB activation and RelB
		Mediators of kidney fibrosis such as Smad3

**Table 3:** Some key answered questions regarding chronodisruption, chronodisruptors and CKD.

When Does Chronodisruption Start in CKD Natural History?	What Are the Key Chronodisruptors in CKD and What Are Their Targets? Can Chronodisruptors Be Targeted Therapeutically?	Other Questions
Before or after the current GFR threshold to define CKD?	Can chronodisruptors be modified by altering the diet or timing of meals?	Is basic research in CKD tainted by chronodisruption resulting from performing mouse and rat experiments during daytime, which should be their inactive period?
Is a decreased GFR needed to trigger CKD-associated chronodisruption?	Or by altering the microbiota?	To what extent the age-associated loss of renal function contributes to age-associated circadian rhythm abnormalities?
Or is pathological albuminuria sufficient to trigger chronodisruption?	Or by drugs modulating their signaling pathways?	
	Does therapeutic targeting of CKD-related chronodisruptors improve outcomes?	
	Has melatonin any role in managing CKD?	
	Has chronopharmacology a role in CKD?	
